# Higher Functional Connectivity of Ventral Attention and Visual Network to Maintain Cognitive Performance in White Matter Hyperintensity

**DOI:** 10.14336/AD.2022.1206

**Published:** 2023-08-01

**Authors:** Xiao Zhu, Ying Zhou, Wansi Zhong, Yifei Li, Junjun Wang, Yuping Chen, Ruoxia Zhang, Jianzhong Sun, Yu Sun, Min Lou

**Affiliations:** ^1^Department of Neurology, the Second Affiliated Hospital of Zhejiang University, School of Medicine, Hangzhou, China.; ^2^Department of Neurology, Zhejiang Hospital, Hangzhou, China.; ^3^Department of Radiology, the Second Affiliated Hospital of Zhejiang University, School of Medicine, Hangzhou, China.; ^4^Key Laboratory for Biomedical Engineering of Ministry of Education of China, Zhejiang University, Zhejiang, China.

**Keywords:** cardiovascular diseases, magnetic resonance imaging, cognition, maintenance

## Abstract

Ventral attention network (VAN), associated with cognitive performance, is one of the functional networks that are most vulnerable in white matter hyperintensity (WMH). Considering the global interaction of networks for cognitive performance, we hypothesized that VAN-related between-network connectivity might play a role in maintaining cognition in patients with WMH. We included 139 participants for both cross-sectional and longitudinal analysis from CIRCLE study (ClinicalTrials.gov ID: NCT03542734) between January 2014 and January 2021. Differences of VAN-related between-network connectivity were compared between normal-cognition (NC) and cognitive-impairment (CI) groups cross-sectionally, and between cognitive-decline (CD) and cognitive non-decline (CND) groups longitudinally by using t-test. False Discovery Rate was used for multiple comparison correction. The relationship between the network connectivity and WMH was tested on linear and quadratic models. Subgroup analysis of different WMH burdens were performed to test the difference of network connectivity between NC and CI groups. Among VAN-related between-network connectivity, only VAN-Visual Network (VN) connectivity was higher both in NC (n = 106) and CND (n = 113) groups versus CI (n = 33) and CD groups (n = 26), respectively. There was an inverted U-shaped relation between periventricular WMH (PWMH) burden and VAN-VN connectivity. Normal-cognition participants had higher VAN-VN connectivity among high, but not low PWMH burden subgroups. These findings suggest that the VAN-VN connectivity plays an important role in maintaining cognitive performance in WMH patients. It may serve as a unique marker for cognitive prediction and a potential target for intervention to prevent cognitive decline in WMH patients.

## INTRODUCTION

White matter hyperintensity (WMH), marked by hyperintense signals in T2-weighted magnetic resonance imaging (MRI) sequences and isointense or hypointense signals in T1-weighted sequences, is one of the key imaging features of cerebral small vessel disease (cSVD) [1]. WMH has been linked to cognitive dysfunction and dementia [2]. However, in clinical practice, there exists a large discrepancy between imaging finding of whole brain WMH burden and cognitive performance [2]. That is, a certain proportion of patients with severe WMH lesion still had intact cognitive performance. This discrepancy raised the need to investigate the underlying neural mechanism of cognitive performance in WMH patients.

Brain functional networks are the segregation of functionally specialized areas [3]. Recently, ventral attention network (VAN) has been highlighted as one of the most important functional networks of interest for cognition [4]. Moreover, VAN was identified as one of the most vulnerable functional networks to WMH [5]. It is well-known that there is a global interaction of networks for cognitive performance. With advanced age or pathological conditions, a more segregated organization of brain networks may be triggered by reduced connectivity between specialized networks [6]. We thus hypothesized that there might be an increased connectivity of functional networks related with VAN in WMH patients to maintain cognitive performance in patients with high WMH burden. It may explain the discrepancy between WMH burden and cognitive performance.

Fortunately, the analysis of between-network functional connectivity has provided a more comprehensive way of depicting the association with cognition. It has been used for a better interpretation of cognitive performance in normal aging, multiple sclerosis, Alzheimer’s disease, *etc*.[7-9]. In summary, we hypothesized that VAN-related between-network connectivity might play a role in maintaining cognition in patients with WMH. Therefore, in the current study, we aimed to investigate the association of VAN-related between-network connectivity with cognitive performance in cross-sectional study, and their association with cognitive non-decline in longitudinal study. Then we further explore the detailed relationship between VAN-related connectivity and WMH lesion burden.

## MATERIALS AND METHODS

### Participants

We retrospectively reviewed the data of consecutive patients recruited in the CIRCLE study (ClinicalTrials.gov ID: NCT03542734) between January 2014 and January 2021. The CIRCLE study is a single-center prospective observational study that enrolls adults (age > 40 years) free of known dementia and stroke, who will undergo neuropsychological test, retinal digital images, and multimodal MRI scan. For current study, the inclusion criterion was patients with WMH features, complete demographics (age, sex, years of education), risk factors (hypertension, hyperlipidemia, diabetes, smoking, and drinking) assessment, baseline, and follow-up (a mean duration of 1.4 ± 0.8 years) MRI scans (including T1, T2 Fluid-attenuated Inversion Recovery (FLAIR), Susceptibility-weighted Imaging (SWI), and resting state-fMRI (rs-fMRI)) and neuropsychological tests. Exclusion criteria included: 1) patients with large head motion during rs-fMRI scan (i.e., mean Power framewise displacement > 0.3 or one of the six head motion parameters > 3 mm/degree); 2) patients with failure of preprocessed fMRI analysis due to failure of T1 segmentation. Concerning follow-up assessment, the majority of the participants in the present study had only one follow-up assessment. For those with multiple times of follow-up assessments, we selected the first follow-up assessment in current study.

### Neuropsychological Assessments

The neuropsychological state, assessed as Mini-Mental State Examination (MMSE) [10] and Montreal Cognitive Assessment (MoCA) [11], was performed by trained physicians (Shenqiang Yan (MD, >10 years of experience) and Yuping Chen (MD, 2 years of experience)) in accordance with the standard protocols. The range of MMSE and MoCA scores were both 0-30 with higher scores indicating better cognitive function. Normal-cognition (NC) based on MMSE was defined as: baseline MMSE raw scores ≥ 27 (years of education ≥ 6), ≥ 24 (0 < years of education < 6), ≥ 21 (years of education = 0), otherwise was defined as cognitive-impairment (CI) [12]. Cognitive decline (CD) based on MMSE was defined when the follow-up MMSE scores decreased at least 3 points than the baseline, otherwise was defined as cognitive non-decline (CND) [13, 14]. Rate of cognitive change based on MMSE was defined as (follow-up MMSE scores - baseline MMSE scores)/duration (years). Normal-cognition based on MoCA (NC_Mo_) was defined as MoCA ≥ 23, otherwise was defined as cognitive-impairment (CI_Mo_) [15]. The definition of Cognitive-decline (CD_Mo_) and cognitive non-decline (CND_Mo_) based on MoCA was the same as in MMSE scores definition.

### MRI Protocol

MRI were acquired on a 3.0 T MR (MR750, GE Healthcare, United States) scanner (partially collected by Jianzhong Sun (MD, > 10 years of experience)). For rs-fMRI, it was acquired using a T2^*^-weighted gradient echo-planar imaging sequence, time of repetition = 2000 ms, time of echo = 30 ms, flip angle = 77°, field of view = 240 × 240 mm^2^, matrix = 64 × 64, slice thickness = 4.0 mm, slice number = 38. For T1 imaging, time of repetition = 8 ms, time of echo = 3 ms, field of view = 250 × 250 mm^2^, matrix size = 250 × 250, inversion time = 450 ms, slice thickness = 1 mm, flip angle = 8°. For T2 FLAIR imaging, time of repetition = 8400 ms, time of echo = 150 ms, field of view = 240 × 240 mm^2^, matrix size = 256 × 256, inversion time = 2100 ms, slice thickness = 4.0 mm, flip angle = 90°.

### Assessment of WMH

WMH was defined as subcortical hyperintensities without cavitation on T2 FLAIR based on the recommendations of Standards for Reporting Vascular Changes on Neuroimaging (STRIVE) [1]. For periventricular WMH (PWMH) score, 0 indicates absence, 1 indicates caps or pencil-thin lining, 2 indicates smooth haloing or thick lining, and 3 indicates irregular periventricular lesions extending into the deep white matter. For deep WMH (DWMH) score, 0 indicates absence, 1 indicates small punctate or nodular lesions, 2 indicates beginning confluent lesions, and 3 indicates confluent lesions [16]. Both PWMH and DWMH burden were set as level 1 (score 0-1), level 2 (score 2), and level 3 (score 3). High PWMH burden was defined as PWMH score of 3 or was defined as low PWMH burden. High DWMH burden was defined as DWMH score ≥ 2 or was defined as low DWMH burden [17]. WMH score was calculated as the sum of PWMH and DWMH score, ranging from 0 to 6. WMH burden was then set as level 1 (score 1-2), level 2 (score 3-4), and level 3 (score 5-6). Especially, high WMH burden was defined as PWMH score of 3 and/or DWMH score ≥ 2, otherwise was defined as low WMH burden[17]. WMH was assessed by Ying Zhou (PhD, 7 years of experience).

### T1 Imaging Processing

T1 imaging was processed by using DPABI[18], which is based on SPM 12 (Wellcome Department of Cognitive Neurology, London, UK) by Xiao Zhu (MD, 2 years of experience). Processing steps included: 1) convert the DICOM format to NIFTI format, 2) skull striping, and 3) gray matter, white matter, and cerebrospinal fluid segmentation by Diffeomorphic Anatomical Registration Through Exponentiated Lie Algebra (DARTEL).

### Rs-fMRI Preprocessing

Rs-fMRI was also preprocessed by using DPABI[18] by Xiao Zhu (MD, 2 years of experience). Preprocessing steps included: 1) convert the DICOM format to NIFTI format, 2) slice timing (removing the first 10 volumes), 3) realignment (to the middle volume), 4) nuisance regression (i.e., Friston-24 head motion parameters, white matter signal and cerebrospinal fluid signal which were extracted from individual T1 segmentation), 5) motion scrubbing (the volume of Power framewise displacement > 0.2 and its previous 1 and post 2 volumes were censored), 6) normalization (by DARTEL to Montreal Neurological Institute space into 3×3×3 mm voxels), 7) smoothing (with a Gaussian kernel of 6 mm), and 8) band-pass filtering (0.01-0.1 Hz).

### Between-network Connectivity

Functional networks were based on a well-established cortical area parcellation atlas derived from rs-fMRI[19]. The Gordon atlas has 286 regions of interest (ROIs) with 12 functional networks (i.e., Auditory network, Cingulo-opercular network, Cingulo-parietal network, Dorsal attention network, Default mode network (DMN), Dorsal somato-motor network, Frontoparietal control network (FPCN), Retrosplenial-temporal network (RN), Salience network, VAN, Ventral somato-motor network, and VN). Detailed number of ROIs for each network were listed on Supplementary Table 5. For each subject, the mean rs-fMRI time series of each 286 ROIs was extracted and correlated with every other ROI’s time course, generating a ROI-to-ROI Pearson correlation matrix (r-matrix). To increase the normality of the data, the r-matrix was converted into z-matrix by using Fisher z-transformation. Between-network connectivity was calculated as the mean ROI-to-ROI z-value between each ROIs of one network and all ROIs of another network. Functional connectivity was calculated by using DynamicBC toolbox [20]. Between-network connectivity was based on an in-house code. Both were conducted in MATLAB R2022a (MathWorks, Inc.).

### Sensitivity Analysis

We did sensitivity analysis on the grouping based on MoCA to test the main results in cross-sectional and longitudinal study. Given the potential confounding effect of low education level (years of education < 6) on MoCA [11, 15, 21], we selected participants with years of education ≥ 6, resulting in 89 participants in total for further analysis.

### Statistical Analysis

All analysis was performed in SPSS packages (IBM, Chicago, version 22.0 for Windows). We checked the normality of all the characteristics by using the Kolmogorov-Smirnov test and histogram inspection. We used Spearman correlation to test the linear correlation between WMH scores and MMSE scores, and Kruskal-Wallis H test to further assess group differences in MMSE scores. All the characteristics were compared between groups using t-tests for continuous variables and chi-squared tests for nominal variables, followed by False Discovery Rate (FDR) correction for multiple comparison correction. Logistic regression model was used to confirm the association between the network connectivity and cognition after adjusting for age, sex, years of education, and WMH scores in cross-sectional study (adjusted for age, sex, and years of education in model 1; added WMH scores in model 2), and adjusting for age, sex, years of education, WMH scores, and baseline MMSE scores in longitudinal study (adjusted for age, sex, and years of education in model 1; added baseline MMSE scores in model 2; further added WMH scores in model 3). To test for relationships between all the characteristics and the rate of cognitive change, t-test was used for nominal variables and Pearson correlation was used for continuous variables. The relation between WMH scores and network connectivity were tested by both linear and quadratic models. A *p* or FDR-corrected *p* (FDR *p*) value < 0.05 was considered to be statistically significant.

**Table 1:** The clinical, demographic, and MRI characteristics of participants.

Characteristics	N = 139
**Demographic and neuropsychological characteristics**	
**Age (mean (SD))**	62.7 (8.9)
**Sex (female%)**	69 (49.6%)
**Years of education (mean (SD))**	8.0 (4.8)
**MMSE (mean (SD))**	26.4 (3.3)
**MRI features**	
**PWMH scores (mean (SD))**	2.0 (0.9)
**DWMH scores (mean (SD))**	2.0 (0.9)
**Risk factors**	
**Hypertension (yes%)**	80 (57.6%)
**Hyperlipidemia (yes%)**	30 (21.6%)
**Diabetes (yes%)**	27 (19.4%)
**Smoking (yes%)**	39 (28.1%)
**Drinking (yes%)**	36 (25.9%)

Note. MMSE, Mini-Mental State Examination; PWMH, periventricular white matter hyperintensity; DWMH, deep periventricular white matter hyperintensity.

## RESULTS

### Clinical, Demographic, and MRI Characteristics

**Figure 1.**
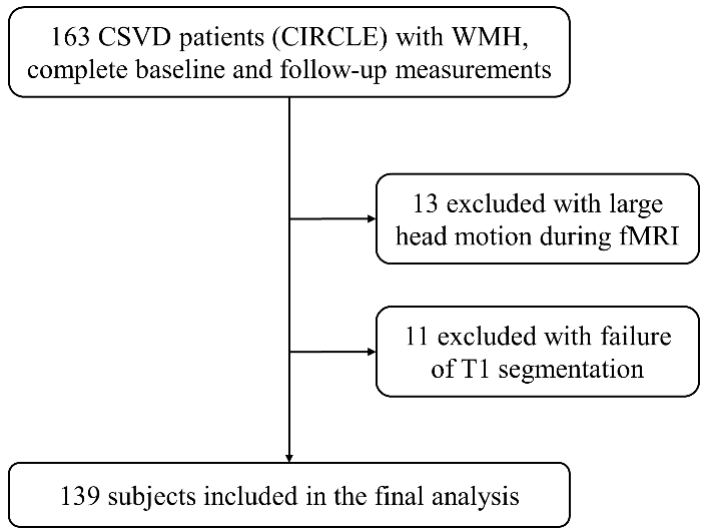
Flow chart visualizing the selection process of participants.

A total of 139 participants were included in the final analysis (Fig. 1), including 106 participants with NC and 33 with CI. Mean age was 62.7 (SD = 8.9) years, and 49.6% (n = 69) of all participants were females. The MMSE scores were 26.4 ± 3.3. PWMH and DWMH scores were both 2.0 ± 0.9. Detailed clinical, demographic, and MRI characteristics were demonstrated in Table 1. Duration between follow-up and baseline was 1.4 ± 0.8 years. There were 113 participants with CND and 26 with CD.

### Association of WMH and Cognition

MMSE scores was not correlated with WMH scores (*ρ* = -0.163, *p* = 0.06), PWMH scores (*ρ* = -0.132, *p* = 0.12) or DWMH scores (*ρ* = -0.162, *p* = 0.06).

**Figure 2.**
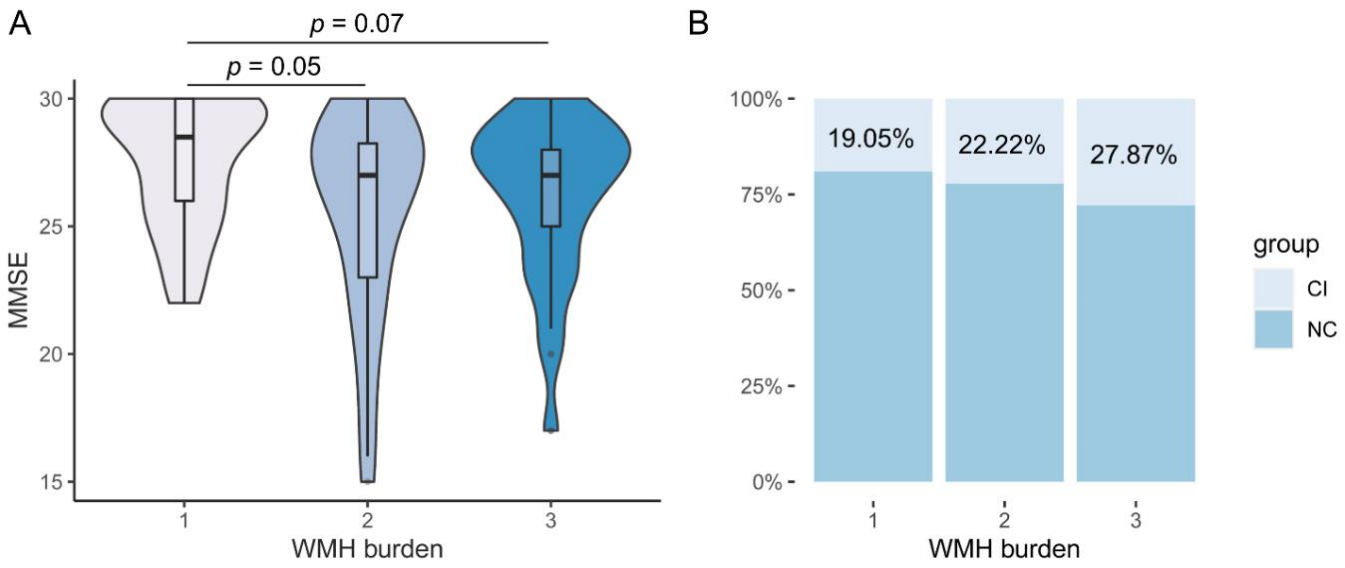
**The association between level of WMH (white matter hyperintensity) burden and cognition**. (**A**) H test showed a difference of MMSE scores in different levels of WMH burden (level 1, n = 42, mean rank = 83.50; level 2, n = 36, mean rank = 62.08; level 3, n = 61, mean rank = 65.38). (**B**) Distribution of cognitive-impairment (CI) and normal-cognition (NC) participants in different levels of WMH burden. There were a large proportion of NC participants in level 3 of WMH burden.

**Figure 3.**
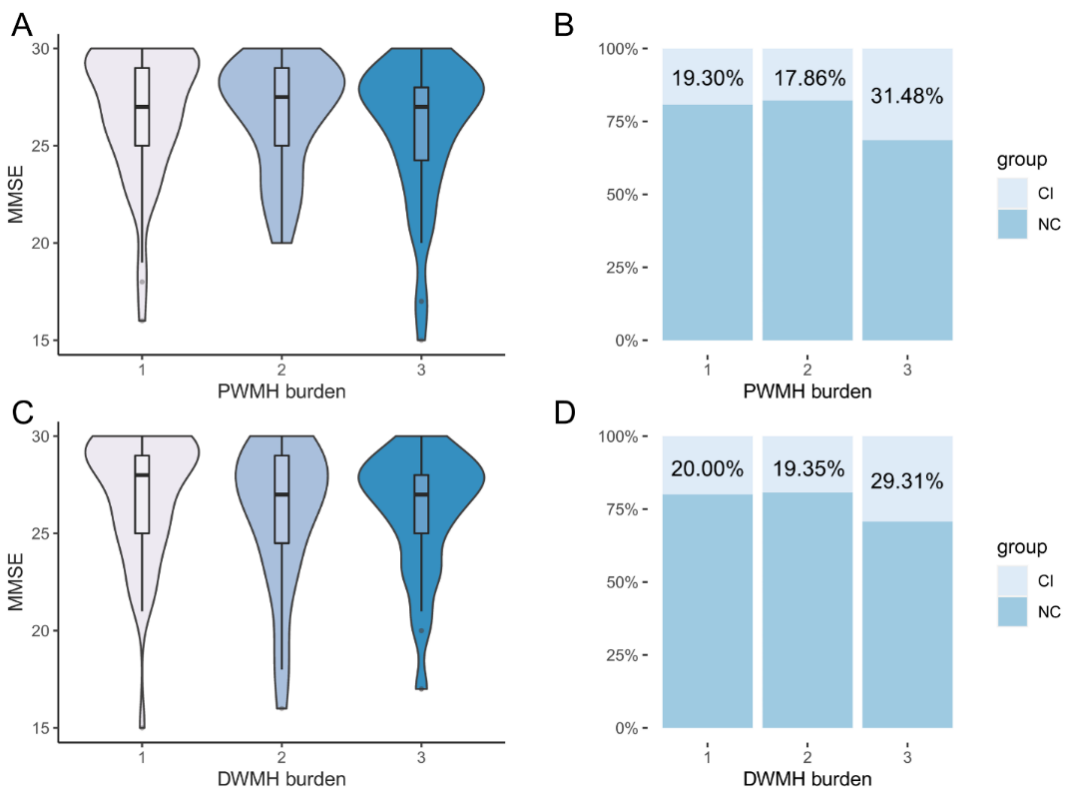
**The association of level of PWMH and DWMH (periventricular and deep white matter hyperintensity) burden with cognition**. (**A, C**) H test showed no difference in different levels of PWMH (level 1, n = 57, mean rank = 76.18; level 2, n = 28, mean rank = 70.04; level 3, n = 54, mean rank = 63.45) and DWMH (level 1, n = 50, mean rank = 79.08; level 2, n = 31, mean rank = 66.26; level 3, n = 58, mean rank = 64.17) burden. (**B, D**) Distribution of cognitive-impairment (CI) and normal-cognition (NC) participants in different levels of WMH burden.

H test showed a difference of MMSE scores among different levels of WMH burden (Fig. 2A, χ^2^ = 7.028, *p* = 0.03). Post-hoc analysis showed near-significant differences of MMSE scores between level 1 and 2 (*p* = 0.05), and between level 1 and 3 (*p* = 0.07), and no difference between level 2 and 3 (*p* > 0.99). However, H test showed no difference of MMSE scores among different levels of PWMH burden (Fig. 3A, χ^2^ = 2.816, *p* = 0.24) or DWMH burden (Fig. 3C, χ^2^ = 4.090, *p* = 0.13).

The distribution of NC and CI participants in different levels of WMH burden (Fig. 2B), PWMH and DWMH burden (Fig. 3B, D) are shown. There were a large proportion of NC participants in level 3 of WMH burden (72.13% (NC) vs 27.87% (CI)).

### Association of VAN-related Between-network Connectivity and Cognition

#### Cross-sectional Study

**Figure 4.**
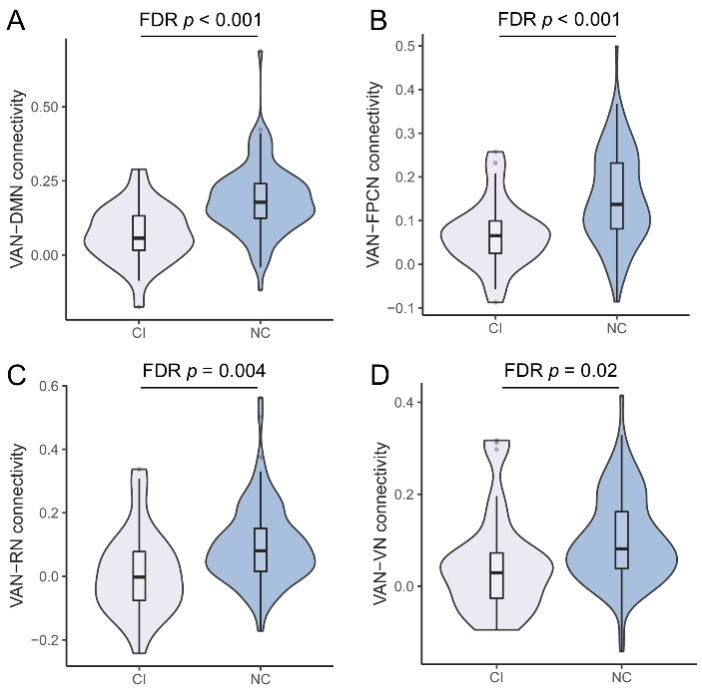
**Violin plot of VAN-related between-network connectivity in cognitive-impairment (CI) (n = 33) and normal-cognition (NC) (n = 106) groups**. (**A**) ventral attention network (VAN)-default mode network (DMN) connectivity. (**B**) VAN-fronto-parietal control network (FPCN) connectivity. (**C**) VAN-retrosplenial-temporal network (RN) connectivity. (**D**) VAN-visual network (VN) connectivity. Violins show the data distribution. Boxes represent the interquartile range, vertical lines show the maximum and minimum values, and horizontal lines indicate the median.

There were no differences in clinical characteristics between NC (n = 106) and CI (n = 33) groups (Table 2, all *p* > 0.05). As for VAN-related between-network connectivity, NC group had higher VAN-DMN (FDR *p* < 0.001), VAN-FPCN (FDR *p* < 0.001), VAN-RN (FDR *p* = 0.004), and VAN-VN (FDR *p* = 0.02) connectivity compared to CI group after FDR correction (Fig. 4, Supplementary Table 1). These associations persisted after adjusting for age, sex, years of education, and WMH scores (Supplementary Table 2, all OR > 1 with *p* ≤ 0.01).

**Table 2:** Comparison of characteristics between CI and NC groups in cross-sectional study and between CD and CND groups in longitudinal study.

	CI group (n = 33)	NC group (n = 106)	*p*	CD group (n = 26)	CND group (n = 113)	*p*
**Demographic characteristics**						
Age (mean (SD))	63.0 (7.5)	62.6 (9.3)	0.84	64.6 (7.7)	62.3 (9.1)	0.22
Sex (female%)	13 (39.4%)	56 (52.8%)	0.18	11 (42.3%)	58 (51.3%)	0.41
Years of education (mean (SD))	7.5 (4.1)	8.2 (5.0)	0.42	6.6 (4.7)	8.4 (4.8)	0.09
**Cognition and follow-up**						
Baseline MMSE scores (mean (SD))	/	/	/	26.7 (3.7)	26.4 (3.2)	0.66
Follow-up MMSE scores (mean (SD))	/	/	/	21.5(5.1)	27.0(3.0)	<0.001
Follow-up duration (mean (SD), years)	/	/	/	1.5 (1.0)	1.3 (0.7)	0.33
**MRI features**						
PWMH scores (mean (SD))	2.1 (1.1)	1.9 (0.9)	0.33	2.0 (1.0)	1.9 (0.9)	0.60
DWMH scores (mean (SD))	2.2 (1.0)	2.0 (0.9)	0.27	2.1 (1.0)	2.0 (0.9)	0.58
**Risk factors**						
Hypertension (yes%)	19 (57.6%)	61 (57.5%)	>0.99	12 (46.3%)	68 (60.2%)	0.19
Hyperlipidemia (yes%)	6 (18.2%)	24 (22.6%)	0.59	2 (7.7%)	28 (24.8%)	0.10
Diabetes (yes%)	5 (15.2%)	22 (20.8%)	0.48	7 (26.9%)	20 (17.7%)	0.28
Smoking (yes%)	13 (39.4%)	26 (24.5%)	0.10	6 (23.1%)	33 (29.2%)	0.53
Drinking (yes%)	12 (36.4%)	24 (22.6%)	0.12	5 (19.2%)	31 (27.4%)	0.39

Note. CI, cognitive-impairment; NC, normal-cognition; CD, cognitive-decline; CND, cognitive non-decline; MMSE, Mini-Mental State Examination; PWMH, periventricular white matter hyperintensity; DWMH, deep periventricular white matter hyperintensity.

#### Longitudinal Study

**Figure 5.**
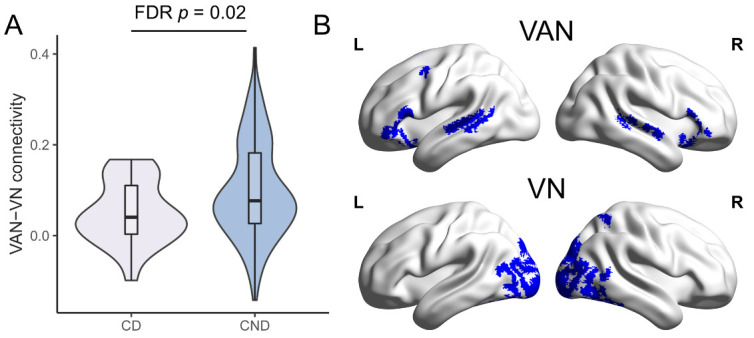
**Comparison of VAN-VN connectivity between cognitive-decline (CD) (n = 26) and cognitive non-decline (CND) (n = 113) groups**. (**A**) Violin plot of ventral attention network (VAN)-visual network (VN) connectivity in CD and CND groups. Violins show the data distribution. Boxes represent the interquartile range, vertical lines show the maximum and minimum values, and horizontal lines indicate the median. (**B**) The distribution of VAN (upper, blue) and VN (lower, blue) were shown in surface view. L = left; R = right.

**Figure 6.**
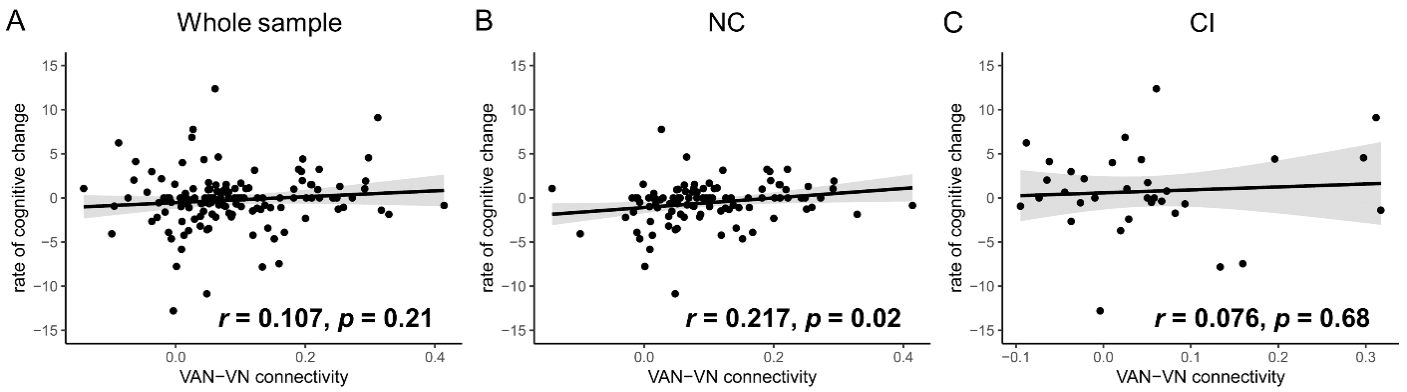
**Correlation between ventral attention network (VAN)-visual network (VN) connectivity and rate of cognitive change**. A) Whole sample (n = 139). B) normal-cognition (NC) group (n = 106). C) cognitive-impairment (CI) group (n = 33). Grey fill indicates 95% Confidence Interval.

There were no differences in clinical characteristics between CD (n = 26) and CND (n = 113) groups (Table 2, all *p* > 0.05). As for VAN-related between-network connectivity, only VAN-VN connectivity was higher in CND group (Fig. 5, Supplementary Table 3, FDR *p* = 0.02), which remained significant after adjusting for age, sex, years of education, baseline MMSE scores, and WMH scores (Supplementary Table 4, OR (95% Confidence Interval) = 6.043e2(2.288-1.596e5), *p* = 0.02). Besides, VAN-VN connectivity had a positive correlation with the rate of cognitive change in NC group (*r* = 0.027, *p* = 0.02), but not in CI group or whole sample (Fig. 6).

### Association of WMH and VAN-VN Connectivity

**Figure 7.**
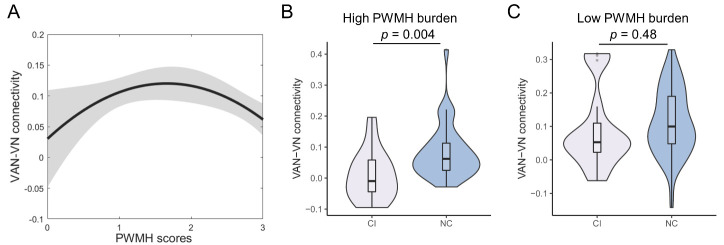
**Association between PWMH scores and VAN-VN connectivity and group comparison in high WMH burden group**. (**A**) Inverted U-shaped relation of periventricular white matter hyperintensity (PWMH) scores and ventral attention network (VAN)-visual network (VN) connectivity (linear model, *β* = -0.147, *p* = 0.08; quadratic model, *β*_1_ = 1.021, *p* = 0.03, *β*_2_ = -1.187, *p* = 0.01) (PWMH = 0, n = 4; PWMH = 1, n = 53; PWMH = 2, n = 28; PWMH = 3, n = 54). Grey fill indicates 95%CI. (**B**) Violin plot of VAN-VN connectivity in cognitive-impairment (CI, n = 17, *M* = 0.008 (*SE* = 0.019)) and normal-cognition (NC, n = 37, *M* = 0.083 (*SE* = 0.014)) groups in high PWMH burden subgroup. (**C**) Violin plot of VAN-VN connectivity in CI (n = 16, *M* = 0.091 (*SE* = 0.030)) and NC (n = 69, *M* = 0.111 (*SE* = 0.012)) groups in low PWMH burden subgroup. Violins show the data distribution. Boxes represent the interquartile range, vertical lines show the maximum and minimum values, and horizontal lines indicate the median.

As Figure 7A shows, there was an inverted U-shaped relation between PWMH scores and VAN-VN connectivity (quadratic model, *β*_1_ = 1.021, *p* = 0.03, *β*_2_ = -1.187, *p* = 0.01) instead of a linear correlation (*β* = -0.147, *p* = 0.08). This relationship was not shown with DWMH (quadratic model, *p* = 0.46) or WMH scores (quadratic model, *p* = 0.22).

#### WMH Burden Subgroup Analysis on Association of VAN-VN connectivity with Cognition

Subgroup analysis of PWMH burden was plotted in Figure 7, while WMH and DWMH burden were in Supplementary Figure 1. In high PWMH burden subgroup, VAN-VN connectivity was higher in NC group (n = 37) than CI group (n = 17) (*p* = 0.004). But in low PWMH burden subgroup, there was no difference of VAN-VN connectivity between CI (n = 16) and NC groups (n = 69) (*p* = 0.48).

Similarly, compared to CI group, NC group had higher VAN-VN connectivity in high WMH (*p* = 0.003) and DWMH (*p* = 0.006) burden subgroup, but not in low WMH (*p* = 0.84) and DWMH (*p* = 0.49) burden subgroup.

### Sensitivity analysis

Cross-sectionally, NC_Mo_ group (n = 63) had higher VAN-VN connectivity than CI_Mo_ group (n = 26) (*M* ± *SE*: 0.111 ± 0.012 versus 0.060 ± 0.024, *p* = 0.04, t-test). Longitudinally, CND_Mo_ group (n = 75) also had higher VAN-VN connectivity than CD_Mo_ group (n = 14) (*M* ± *SE*: 0.105 ± 0.012 versus 0.045 ± 0.023, *p* = 0.05, t-test). These were consistent with the results derived from the definition based on MMSE.

## DISCUSSION

The main findings of the current study include: 1) Higher VAN-VN connectivity was associated with normal cognition cross-sectionally, and with cognitive non-decline longitudinally. 2) There was an inverted U-shaped relation between VAN-VN connectivity and PWMH burden. 3) Normal-cognition participants had higher VAN-VN connectivity among high, but not low WMH, PWMH, and DWMH burden subgroup than participants with cognitive impairment. All these findings revealed that higher VAN-VN connectivity was involved in cognitive maintenance in normal-cognition and cognitive non-decline participants with WMH lesion.

Different from previous studies, our analysis focused on the underlying neural mechanism of normal-cognition and cognitive non-decline in patients with WMH lesion. VAN has been identified as one of the most important functional networks of interest for cognition. In Alzheimer’s disease, the temporal dynamics of VAN was correlated with global cognition[4]. VAN disturbance, measured by eigenvector centrality, was also associated with cognitive impairment in multiple sclerosis[22]. Similarly, Crockett et al.[5] found that the WMH volume in VAN was negatively correlated with global cognition. However, all these studies only observed the characteristics of the single network, which could not fully explain the preservation of cognition in WMH patients. In response to detrimental brain changes, the engagement of a more integrated organization of networks may be triggered to counteract cognitive decline. Thus, the identification of between-network functional connectivity in our study is important to explore the underlying cognitive abilities that are preserved or declined.

Our study also suggested a great individual variability in cognitive trajectories of WMH patients, similar to healthy ageing. There was a trend-level correlation between WMH burden and cognition in our patients which may be due to the small sample size. More important, the distribution of normal-cognition and cognitive impairment participants demonstrated a discrepancy between the severity of WMH and cognitive performance. It is not controversial with the view that WMH had a negative impact on cognitive performance[23, 24]. Rather, it complemented this view by adding the concept of “discrepancy” especially in high WMH burden patients.

We found that higher VAN-VN connectivity was associated with preserved cognitive performance. VAN is involved in orienting attention to unexpected stimulus[25], while VN participates in the perception and processing of the visual stimulus[26]. VAN is found to be deactivated during tasks requiring continued attention and activated if an unexpected stimulus appears[27, 28], suggesting its close relationship with VN. Collectively, higher resting-state VAN-VN connectivity may indicate a more alerting state in preparation for upcoming stimuli. It may relate to cognitive maintenance. Maintenance was referred to the preservation of neural resources with a balance of repair and neural deterioration[29]. Higher VAN-VN connectivity may indicate a full resistance of repair in reaction to WMH lesion, while lower connectivity may be insufficient to offset brain damage and gradually lead to cognitive dysfunction. During face-name associative encoding, cognitive maintenance occurred when hippocampus activity in the old cognitive maintainers was higher than that in the old decliners, but comparable to the young adults[30]. However, our novel finding needs to be confirmed in further study.

Intriguingly, an inverted U-shaped relation between VAN-VN connectivity and PWMH burden was observed, which may indicate a PWMH burden-dependent effect of repair. Particularly, patients with high PWMH burden had a damaged repair process in cognitive maintenance indicated by lower VAN-VN connectivity compared to patients with relatively low PWMH burden. Furthermore, our finding that normal-cognition participants had higher VAN-VN connectivity among high PWMH burden subgroup, indicated the heterogeneity of the repair efficacy associated with cognitive maintenance. On the contrary, in low PWMH burden subgroup, the level of cognitive maintenance was regardless of the repair capacity [29]. All these results indicate that the overall efficacy of cognitive maintenance depends both on the burden of PWMH lesion and the efficacy of connectivity repair.

In cross-sectional study, except for VAN-VN connectivity, VAN-DMN, VAN-FPCN, and VAN-RN connectivity were also associated with normal-cognition. Previous studies found that DMN, FPCN, and RN were all associated with cognition[31-33]. As VAN participates in stimulus-driven attention allocation[27, 28], higher VAN-DMN, VAN-FPCN, and VAN-RN connectivity may indicate more efficient stimulus-driven cognitive processing. However, longitudinal analysis did not replicate these results in association with cognitive decline. It may be due to the small sample size, or cross-sectional study simply reflects a covarying relation between connectivity and cognition.

Strengths of the study include the combination of cross-sectional and longitudinal studies in investigating the association between VAN-related between-network connectivity and cognition, with the exploration of the neural mechanism underlying the association. Considering the repair role of VAN-VN connectivity in cognitive maintenance related to WMH, VAN-VN connectivity may serve as a potential functional marker to predict cognitive decline in WMH patients. A future study is needed to prospectively validate the application value of VAN-VN connectivity in routine clinical practice to identify WMH patients at risk of cognitive decline. Moreover, our findings highlight individuals with lower VAN-VN connectivity who are in urgent need of early intervention. Nowadays, transcranial magnetic stimulation (TMS), and transcranial direct or alternate current stimulation (tACS) have been proven to be effective in improving the cognitive performance by targeting the relevant brain regions directly[34, 35]. For example, in mild cognitive impairment (MCI), tACS was effective in facilitating cognitive function by increasing beta activity of dorsolateral prefrontal cortex (DLPFC)[35]. Similarly in MCI, high frequency TMS was proved to improve Trail-Making Test performance when targeting inferior frontal gyrus which was engaged in attention function[36]. By delivering simultaneous in-phase tACS on DLPFC and temporoparietal junction, the auditory hallucination was improved in schizophrenia, which further suggested that tACS is potential as a network-level approach to modulate neural oscillations related to clinical symptoms[37]. Apart from direct magnetic and electric stimulation on the loop, an individualized neurofeedback program may also be helpful in enhancing the VAN-VN connectivity by self-regulation when receiving visual or auditory stimuli of the connectivity to mitigate cognitive decline. Neurofeedback is proven useful and widely adopted in cognitive and clinical neuroscience [38]. Therefore, our finding provides a neural basis of the intervention by using these non-invasive methods to maintain cognitive performance in WMH patients in the future.

There are some limitations to be considered. First, though a close association was found for VAN-VN connectivity and cognition, direct evidence is still lacking to prove their causality. Intervention with electric or magnetic stimulation on VAN-VN connectivity in a randomized control trial is warranted. Second, our sample size was relatively small in comparison to other neuroimaging studies. Third, the included participants had higher MMSE scores, higher burden of WMH and higher rates of hypertension, hyperlipidemia, and drinking than excluded participants in CIRCLE study. There might be selection bias due to the different compliance of the participants, which should be carefully addressed in future studies. Fourth, the follow-up duration of our cohort was heterogeneous. Though there was not significant difference of the duration between CD and CND group in longitudinal study, it should be carefully concerned in future studies. Lastly, MMSE scores only reflect the global cognition, therefore a more comprehensive neuropsychological battery is necessary to clarify the specific cognitive domain. Future studies with a broader range of age and larger sample size are warranted to verify the role of VAN-VN connectivity in the more specific domain of cognitive performance. Apart from VAN which is the main concern in the present study, future study with advanced methods (e.g., network gradient and multilayer network connectivity) could be used to explore whether other networks or their characteristic is associated with cognitive performance in patients with WMH.

In conclusion, we reported that higher VAN-VN connectivity may indicate more efficient repair to WMH lesion in maintaining cognitive performance. This stresses the importance of integrating rs-fMRI with other imaging modalities to improve the identification of WMH patients who are at higher risk in impending cognitive decline.

## Supplementary Materials

The Supplementary data can be found online at: www.aginganddisease.org/EN/10.14336/AD.2022.1206.
