# Supplementary material for: Higher Functional Connectivity of Ventral Attention and Visual Network to Maintain Cognitive Performance in White Matter Hyperintensity
Source: Aging Dis. 2023 Aug 1;14(4):1472–82. doi: 10.14336/AD.2022.1206 (PMC10389834; doi:10.14336/AD.2022.1206)
Supplement: Supplementary file 1 — The Supplementary data can be found online at: www.aginganddisease.org/EN/10.14336/AD.2022.1206. [file AD-14-4-1472-s.pdf]

SUPPLEMENTARY DATA

Higher Functional Connectivity of Ventral Attention and Visual Network to Maintain Cognitive Performance in White Matter Hyperintensity

Xiao Zhu[1], Ying Zhou, Wansi Zhong, Yifei Li, Junjun Wang, Yuping Chen, Ruoxia Zhang, Jianzhong Sun, Yu Sun, Min Lou

© 2022. Zhu X et al. Published online at https://www.aginganddisease.org/EN/10.14336/AD.2022.1206

SUPPLEMENTARY DATA

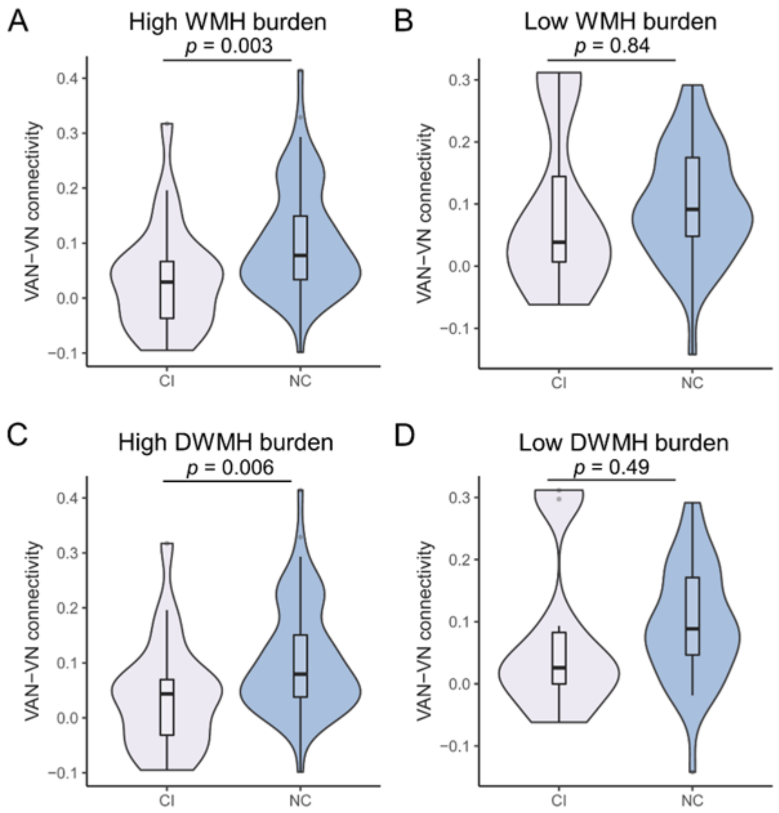

Supplementary Figure 1. Comparison of ventral attention network (VAN)-visual network (VN) connectivity between cognitive-impairment (CI) and normal-cognition (NC) groups in different subgroups. A) Violin plot of high WMH burden subgroup analysis in CI (n = 25, *M* = 0.035 (*SE* = 0.019)) and NC (n = 67, *M* = 0.103 (*SE* = 0.012)) groups. B) Violin plot of low WMH burden subgroup analysis in CI (n = 8, *M* = 0.090 (*SE* = 0.049)) and NC (n = 39, *M* = 0.098 (*SE* = 0.014)) groups. C) Violin plot of high DWMH burden subgroup analysis in CI (n = 23, *M* = 0.038 (*SE* = 0.020)) and NC (n = 66, *M* = 0.104 (*SE* = 0.012)) groups. D) Violin plot of low DWMH burden subgroup analysis in CI (n = 10, *M* = 0.071 (*SE* = 0.041)) and NC (n = 40, *M* = 0.095 (*SE* = 0.014)) groups. Violins show the data distribution. Boxes represent the interquartile range, vertical lines show the maximum and minimum values, and horizontal lines indicate the median.

Supplementary Table 1. Comparison of VAN-related between-network connectivity between CI and NC groups.

	CI group (n=33) *	NC group (n=106) *	*p*	FDR *p*
VAN-AN	0.179 (0.020)	0.190 (0.012)	0.66	0.72
VAN-CO	0.102 (0.016)	0.105 (0.009)	0.85	0.85
VAN-CP	0.016 (0.016)	0.063 (0.012)	0.05	0.10
**VAN-DMN**	**0.070 (0.017)**	**0.185 (0.011)**	**< 0.001**	**< 0.001**
VAN-DAN	0.042 (0.012)	0.056 (0.009)	0.42	0.52
**VAN-FPCN**	**0.068 (0.013)**	**0.151 (0.010)**	**< 0.001**	**< 0.001**
**VAN-RN**	**0.010 (0.023)**	**0.096 (0.012)**	**0.001**	**0.004**
VAN-vSM	0.001 (0.013)	0.018 (0.011)	0.43	0.52
VAN-dSM	0.048 (0.019)	0.095 (0.014)	0.05	0.10
VAN-SN	0.128 (0.020)	0.149 (0.011)	0.36	0.52
**VAN-VN**	**0.048 (0.019)**	**0.101 (0.009)**	**0.008**	**0.02**

Note. CI, cognitive-impairment; NC, normal-cognition; AN, auditory network; CO, cingulo-opercular network; CP, cingulo-parietal network; DMN, default mode network; DAN, dorsal attention network; FPCN, fronto-parietal control network; RN, retrosplenial-temporal network; vSM, ventral somatomotor network; dSM, dorsal somatomotor network; SN, salience network; VAN, ventral attention network; VN, visual network.
* Data in paratheses are SE.

SUPPLEMENTARY DATA

Supplementary Table 2. Logistic regression analysis for normal cognition.

	OR (95% Confidence Interval)	*p*
Model 1: adjusting for age, sex, and years of education		
VAN-DMN	1.20e5 (5.97e2-2.41e7)	< 0.001
VAN-FPCN	2.68e4 (1.12e2-6.42e6)	< 0.001
VAN-RN	5.37e2 (9.63-2.99e4)	0.002
VAN-VN	4.23e2 (3.69-4.85e4)	0.01
Model 2: adjusting for age, sex, years of education, and WMH scores		
VAN-DMN	1.16e5 (5.80e2-2.30e7)	< 0.001
VAN-FPCN	2.66e4 (1.12e2-6.32e6)	< 0.001
VAN-RN	5.37e2 (9.53-3.03e4)	0.002
VAN-VN	3.96e2 (3.41-4.58e4)	0.01

Note. VAN, ventral attention network; DMN, default mode network; FPCN, fronto-parietal control network; RN, retrosplenial-temporal network; VN, visual network; WMH, white matter hyperintensity.

Supplementary Table 3. Comparison of VAN-related between-network connectivity between CD and CND groups.

	CD group (n=26) *	CND group (n=113) *	*p*	FDR *p*
VAN-DMN	0.138 (0.020)	0.162 (0.012)	0.36	0.69
VAN-FPCN	0.120 (0.023)	0.134 (0.010)	0.52	0.69
VAN-RN	0.066 (0.022)	0.078 (0.012)	0.69	0.69
**VAN-VN**	**0.050 (0.013)**	**0.098 (0.010)**	**0.005**	**0.02**

Note. CD, cognitive-decline; CND, cognitive non-decline; VAN, ventral attention network; DMN, default mode network; FPCN, fronto-parietal control network; RN, retrosplenial-temporal network; VN, visual network.
* Data in paratheses are SE.

Supplementary Table 4. Logistic regression analysis for cognitive non-decline.

	OR (95% Confidence Interval)	*p*
Model 1: adjusting for age, sex, and years of education		
VAN-VN	1.640e2 (0.993-2.710e4)	0.05
Model 2: adjusting for age, sex, years of education, and baseline MMSE		
VAN-VN	6.035e2 (2.288-1.592e5)	0.02
Model 3: adjusting for age, sex, years of education, baseline MMSE, and WMH scores		
VAN-VN	6.043e2 (2.288-1.596e5)	0.02

Note. VAN, ventral attention network; VN, visual network; MMSE, Mini-Mental State Examination; WMH, white matter hyperintensity.

© 2022. Zhu X et al. Published online at https://www.aginganddisease.org/EN/10.14336/AD.2022.1206

Supplementary Table 5. The number of ROIs for each of the 12 functional networks

Network	AN	CO	CP	DMN	DAN	FPCN	RN	vSM	dSM	SN	VAN	VN
Number	24	40	5	41	32	24	8	8	38	4	23	39

Note. AN, auditory network; CO, cingulo-opercular network; CP, cingulo-parietal network; DMN, default mode network; DAN, dorsal attention network; FPCN, fronto-parietal control network; RN, retrosplenial-temporal network; vSM, ventral somatomotor network; dSM, dorsal somatomotor network; SN, salience network; VAN, ventral attention network; VN, visual network; ROIs, regions of interest.

Supplementary Table 6. The comparison of clinical, demographic, and MRI characteristics between included and excluded participants.

Characteristics	Included (N = 139)	Excluded (N = 1018)	*p*
Demographic and neuropsychological characteristics			
Age (mean (SD))	62.7 (8.9)	61.4 (8.3)	0.10
Sex (female%)	69 (49.6%)	452 (44.4%)	0.24
Years of education (mean (SD))	8.0 (4.8)	8.3 (4.0)	0.57
MMSE (mean (SD))	26.4 (3.3)	25.5 (4.3)	**0.002**
MRI features			
PWMH scores (mean (SD))	1.9 (0.9)	1.7 (0.9)	**0.001**
DWMH scores (mean (SD))	2.0 (0.9)	1.5 (1.0)	**< 0.001**
Risk factors			
Hypertension (yes%)	80 (57.6%)	672 (66.0%)	**0.05**
Hyperlipidemia (yes%)	30 (21.6%)	327 (32.1%)	**0.01**
Diabetes (yes%)	27 (19.4%)	198 (19.4%)	> 0.99
Smoking (yes%)	39 (28.1%)	363 (35.7%)	0.08
Drinking (yes%)	36 (25.9%)	438 (43.0%)	**< 0.001**

Note. MMSE, Mini-Mental State Examination; PWMH, periventricular white matter hyperintensity; DWMH, deep periventricular white matter hyperintensity.

© 2022. Zhu X et al. Published online at https://www.aginganddisease.org/EN/10.14336/AD.2022.1206
